# Transcriptional Profiling of Human Monocytes Identifies the Inhibitory Receptor CD300a as Regulator of Transendothelial Migration

**DOI:** 10.1371/journal.pone.0073981

**Published:** 2013-09-18

**Authors:** Sharang Ghavampour, Carsten Lange, Cristina Bottino, Volker Gerke

**Affiliations:** 1 Center for Molecular Biology of Inflammation, Institute of Medical Biochemistry, Muenster, Germany; 2 Dipartimento di Medicina Sperimentale, Sezione di Patologia Generale, Laboratorio di Immunologia Clinica e Sperimentale, Instituto Giannina Gaslini, Genova, Italy; Keio University School of Medicine, Japan

## Abstract

Local inflammatory responses are characterized by the recruitment of circulating leukocytes from the blood to sites of inflammation, a process requiring the directed migration of leukocytes across the vessel wall and hence a penetration of the endothelial lining. To identify underlying signalling events and novel factors involved in these processes we screened for genes differentially expressed in human monocytes following their adhesion to and passage through an endothelial monolayer. Functional annotation clustering of the genes identified revealed an overrepresentation of those associated with inflammation/immune response, in particular early monocyte to macrophage differentiation. Among the gene products so far not implicated in monocyte transendothelial migration was the inhibitory immune receptor CD300a. CD300a mRNA and protein levels were upregulated following transmigration and engagement of the receptor by anti-CD300a antibodies markedly reduced monocyte transendothelial migration. In contrast, siRNA mediated downregulation of CD300a in human monocytes increased their rate of migration. CD300a colocalized and cosedimented with actin filaments and, when activated, caused F-actin cytoskeleton alterations. Thus, monocyte transendothelial migration is accompanied by an elevation of CD300a which serves an inhibitory function possibly required for termination of the actual transmigration.

## Introduction

The inflammatory response of vascularized tissue to perturbations such as injury and infection is characterized by the recruitment of circulating leukocytes to the actual site of perturbation. In order to reach these sites, leukocytes have to penetrate the vessel wall in a directional manner. This process is governed by a cascade of interactions between leukocyte and endothelial cell surface molecules. Following inflammatory stimulation leukocytes engage in initial, primarily selectin-mediated interactions with the activated endothelium that typically lead to tight adhesive contacts mediated by integrins, whose activity is increased upon stimulation of leukocyte chemokine receptors. Finally leukocytes transverse the endothelial blood vessel lining through a paracellular, in some cases also transcellular, route that requires the activity of several endothelial adhesion molecules such as PECAM, CD99, VE-cadherin and JAM family proteins (for reviews see [1]–[5]).

Different signaling pathways are triggered in migrating leukocytes upon interaction with endothelial cells (for reviews see [2], [4], [6], [7]). Activation of these signaling cascades is associated with leukocyte cell polarization and directional movement, which collectively support migration on and finally through the endothelial cell layer. In addition, the engagements of integrins and other adhesion receptors have been linked to the regulation of leukocyte gene expression and mRNA stability [8], [9]. The genes affected range from transcription factors, metabolic enzymes and kinases to cell adhesion molecules. Some of these gene expression changes contribute to leukocyte differentiation [10] and, consequently, leukocyte functions in the inflamed tissue (for an overview see [11]). Gene expression changes have also been analyzed specifically in monocyte subpopulations following activation of inflammatory migration. Such expression profiles revealed an upregulation of certain chemoattractants, proteases possibly required for migration and pattern recognition receptors [12]. Furthermore, following adhesion to naive endothelium primary monocytes have been shown to undergo significant gene expression changes towards a more differentiated phenotype [13].

To extend these observations we sought to obtain a comprehensive overview of monocyte gene expression regulation during the process of transendothelial migration. This was achieved by recording global gene expression profiles of transmigrated versus stationary control monocytes and led to the identification of 81 up- and 75 downregulated mRNAs showing an expression change of at least 1.8 fold. CD300a was among the upregulated genes. It belongs to the group of inhibitory immune receptors that can act as negative regulators counteracting excessive inflammation. Inhibitory receptors have been first characterized on cytotoxic T cells and natural killer (NK) cells because of their ability to terminate the cytotoxic activity of these cells [14], [15]. CD300a is a 60-KD type I transmembrane glycoprotein containing a single extracellular Ig domain and three classical ITIM motifs in its cytoplasmic tail. It is expressed in NK cells and cytotoxic T cells but also in monocytes and granulocytes [16]. Monoclonal antibody-mediated crosslinking had been used to activate the receptor and study functional aspects. In NK cells, such CD300a crosslinking resulted in an inhibition of cytotoxic activity via tyrosin phosphorylation of ITIM motifs and a subsequent recruitment of SHP-1 and SHP-2 phosphatases [16]. In mast cells, CD300a engagement inhibited IgE dependent mast cell activity and SCF mediated survival by recruiting SHP-1 and SHIP [17]. An inhibitory role of CD300a was also observed in eosinophils where antibody-engagement of the receptor suppressed the effect of eotaxin, IL-5, and GM-CSF by recruiting the SHP-1 phosphatase [18]. Furthermore, CD300a can modulate the function of some activating receptors such as CD32a in neutrophils [19] and kit in mast cells [20]. Here, we show that an inhibitory role of CD300a also extends to monocyte transendothelial migration since this process is inhibited by antibody-engagement of the receptor. Thus, upregulation of CD300a during transendothelial migration appears to represent a means of fine-tuning monocyte activity and counteracting the migratory stimulation possibly following the actual transendothelial passage.

## Materials and Methods

### Cell culture and primary cell isolation, DNA constructs and siRNAs

HMEC-1 cells were cultivated at 37°C and 3% CO_2_ in MCDB-131 medium supplemented with 10% FCS Gold, 20 mM L-glutamine, 50 µg/ml gentamycin, 10 ng/ml epidermal growth factor and 10 µg/ml hydrocortisone.

Human peripheral blood monocytes were isolated from buffy coats [21]. In brief, density centrifugation was performed using the Ficoll separation solution with a density of 1,077 g/ml (PAA Laboratories, Germany) to isolated mononuclear cells. In a second round of centrifugation, monocytes were enriched using Percoll separation solution with a density of 1,139 g/ml (Amersham Biosciences, Germany). After washing, the cells were cultured overnight in McCoy's 5A medium supplemented with 15%FCS, pencillin/streptomycin and 2 mM L-Glutamine in Teflon-coated bags to prevent adherence and subsequent activation. Monocyte purity was routinely >80% when assessed by counting in a Coulter Counter Z2 (Coulter, Krefeld, Germany).

For ectopic expression full length cDNA encoding CD300a (imaGenes) was cloned into pcDNA3.1 (–) (Invitrogen) and pEGFP-N3 (Clontech) expression vectors. CD300a knock-down experiments employed freshly isolated monocytes (2×10^7^/100 µl) that were transfected with specific Stealth siRNA oligonucleotides directed at CD300a (5′-UCA CUG CGG CCA AGA CCU CAA CAA U-3′) or control siRNAs (Invitrogen) using the Amaxa transfection method (Lonza, Germany). Transmigration was assessed two days after transfection using the assay described below.

### Transmigration assay

HMEC-1 cell monolayers were employed in monocyte transmigration analyses as described previously [22]. Briefly, 2,2×10^5^ HMEC-1 cells were seeded on fibronectin-coated transwell filters with a 5 µm pore size (Corning) and grown to confluency. After 48 hours, medium and non-adherent cells were removed and 600 µl assay medium (DMEM with 4,5 g/l glucose supplemented with 10% FCS Gold and 20 mM L-glutamine) were added to the lower compartment of a two chamber system separated by the transwell filters. 2×10^6^ monocytes in 100 µl assay medium were added to the upper chamber, and cells were subsequently incubated at 37°C and 5% CO_2_ for 4 hours. Cells that had transmigrated through the endothelial monolayer were recovered in the lower culture chamber and counted in a Z2 Coulter Counter. To verify the integrity of the endothelial monolayer after the assay, the upper chamber was washed twice with phosphate-buffered saline (PBS), stained with DiffQuick (Dade Behring, Düdingen, Switzerland), air-dried, and mounted on glass slides for microscopic analysis. CD300a antibody-engagement experiments were carried out by incubation of freshly isolated monocytes (2×10^6^/100 µl) with 15 µg/ml of the anti-CD300a E59.126 [16] or matched control antibodies (anti-cmyc, Exalpha Biologicals; and anti-p58.2, Abcam) at 37°C, 5% CO_2_ for 30 minutes. Afterwards cells were subjected to the migration assay.

### DNA microarray analysis

Microarray hybridization was carried out with monocyte mRNA from three independent transmigration experiments. In each experiment, transmigrated monocytes were compared to monocytes from the same preparation kept for the same time under identical conditions in tissue culture dishes in the absence of endothelial cells. Total cellular RNA was then isolated from migrated and control monocytes using the RNeasy kit (Qiagen, Hilden, Germany). RNA was quantified by UV spectrophotometry and integrity was verified by agarose gel electrophoresis. Samples for microarray hybridisation were prepared according to the manufacturer's instructions and 10 µg of fragmented cRNA were hybridised to Affymetrix Human Genome U95Av2 Gene Chip arrays according to the manufacturer's instructions (Affymetrix, Santa Clara, CA). Arrays were washed and stained using the Gene Chip Fluidics station 400 (Affymetrix). Fluorescence signals were recorded by the HP G2500A Gene Array Scanner (Affymetrix) and data were processed by MicroArray suite (MAS) software 5.0 (Affymetrix).

### Real-time quantitative PCR

RNA was isolated from transmigrated monocytes or non-migrated monocytes as described above. Reverse transcription was performed using 6 µg of total RNA and 100 pmol/µl of a T7-(dT)_ 24_-primer (5′-GCCAGTGAATTGTAATACGACTCACTATAGGGAGGCGGTTTTTTTTTTTTTTTTTTTTTTTT-3′). The cDNA was subjected to real-time PCR using an ABI prism 7900 HT RT-PCR system. Primer sequences used for the amplification of the genes selected are given in Table 1. Expression level of all genes were normalized to GAPDH controls amplified with oligonucleotides 5′-CTTCATTGACCTCAACTACATG-3′ (sense) and 5′-TGTCATGGATGACCTTGGCCCCAG-3′ (antisense) using the 2^−ΔΔC(T)^-method [23].

**Table 1:** Primers used for real time RT-PCR.

Accession	Gene symbol	Description	Primer sequence (5′–3′)
AI687419	AI687419	AI687419	GGAGAATGGAACCAAGTTGGAA [s]
			TGGAGTTGCTCTTCTTGAGGAGTA [a]
U83171	CCL22	chemokine (C-C motif) ligand 22	GCGTGGTGTTGCTAACCTTCA [s]
			AAGGCCACGGTCATCAGAGTAG [a]
AF020314	CD300A	CD300A antigen	CTTTGTCAATCACAGCCCCATAG [s]
			AGGTGAACAGATCTCCGCTTTG [a]
L31584	CCR7	chemokine (C-C motif) receptor 7	GATGAGGTCACGGACGATTACAT [s]
			GGCCCACGAAACAAATGATG [a]
U03057	FSCN1	fascin homolog 1, actin-bundling protein (Strongylocentrotus purpuratus)	AGCAAGAATGCCAGCTGCTACT [s]
			TCGCAGAACTCGAAGAAGAAGTC [a]
M60974	GADD45A	growth arrest and DNA-damage-inducible, alpha	AAGACCGAAAGGATGGATAAGGT [s]
			GCTTGGCCGCTTCGTACA [a]
U82278	LILRA2	leukocyte immunoglobulin-like receptor, subfamily A, member 2	ATACAAGAGCCTGGGAAGAATGG [s]
			TCAAATGCCACCTGTGAGACA [a]
Z14138	MAP3K8	mitogen-activated protein kinase kinase kinase 8	TCAGCCACGCTGTCAGAGTCT [s]
			GGTCCCCGAACAAGATTGAA [a]
X55740	NT5E	5′-nucleotidase, ecto (CD73)	CCCTCTCAAAATGGACGAGGTA [s]
			TCTTGGTCACCAGAGTCATGTCTT [a]
AB023209	PALLD	palladin	GCTTGTGGTTGCTGCTAAAGAA [s]
			TCGGTCAGTGCTGTGAGTGAGT [a]
S67334	PIK3CB	phosphatidylinositol 3-kinase p110 beta isoform = 110 kda catalytic subunit	CTAATGTGTCAAGTCGAGGTGGAA [s]
			GGAAAATCTCTCGGCAGTCTTGT [a]
AJ001014	RAMP1	receptor (calcitonin) activity modifying protein 1	GAAGAGCTCACAGGAGTCCAGAGTA [s]
			CCCTTTTAATCACAAACCACTCATC [a]
L13463	RGS2	regulator of G-protein signalling 2, 24kDa	TGGAAGACCCGTTTGAGCTACT [s]
			GCAGCTCGTCAAATGCTTCTG [a]
AJ132099	VNN1	vanin 1	TGAGCATGCAGCGATATTGC [s]
			TGATGTGATCGCTCCTTCCA [a]

### Transfection and immunostaining of COS-7 cells

COS-7 cells were transfected with the full length CD300a construct using Effectene (Qiagen). Two days after transfection, cells were treated with 5 μg/ml anti-CD300a antibodies and incubated for 20 minutes. In some experiments, 4 μM cytochalasin D (Calbiochem) were added to the cells for 30 minutes to disrupt the actin cytoskeleton. Cells were then fixed with 4% formaldehyde, permeabilized with 0.5% Triton X-100 and treated with 3% BSA in PBS for 1 h at room temperature. Staining employed Alexa-Fluor 488 anti-mouse antibodies (Dianova) to detect surface-associated anti-CD300a antibodies and Phalloidin-TRITC (Sigma) to label F-actin. Immunostained cells were analysed with a LSM 510 META Laser Scanning Microscope (Zeiss).

### Actin-sedimentation assay

COS-7 cells ectopically expressing full length CD300a were lysed in 0.5% Triton X-100, 10 mM Tris/HCl, pH 7.4, 1 mM MgCl_2_ with protease inhibitors and centrifuged at 400×g for 30 minutes. Supernatants were collected into new tubes, treated with 10 μM nocodazole (Sigma) to disrupt microtubules and subjected to ultracentrifugation (50,000×g, 5 minutes) to pellet actin filaments. Pellets were resuspended in SDS-sample buffer and, together with samples of the total lysates, were subjected to immunoblot analysis using anti-CD300a (E59.126) and anti-actin antibodies.

### Immunofluorescence of monocytes

Freshly isolated monocytes were pre-incubated with anti-CD300a antibodies (E59.126, 5–15 μg/ml) or isotype matched control antibodies (anti-cmyc; Exalpha Biologicals, 5–15 μg/ml) for 10 minutes at 37°C, 5% CO_2_. Thereafter, the cells were seeded on coverslips and incubated for another 30 minutes, fixed with methanol (−20°C) and stained with anti-CD300a antibodies followed by Alexa-Fluor 488 anti-mouse antibodies (Dianova) and Phalloidin-TRITC. Immunostained cells were analysed with a LSM 510 META Laser Scanning Microscope.

### Adhesion assay

Monocytes (1×10^5^) were treated with LPS (5 μg/ml), anti-LFA-1 (20 μg/ml, Thermo scientific), anti-c-myc or anti-CD300a antibodies (5 μg/ml each) and then added to confuently grown HMEC-1 followed by incubation at 37°C. Non-adherent cells were removed by washing with PBS containing Ca++/Mg++ and the amount of adherent cells was determined by measuring myeloperoxidase activity [22] . In order to exclude an effect of anti-CD300a antibodies on monocytes adhesion, integrin activation following antibody treatment was analysed using a monoclonal antibody (9EG7) that recognizes the activated form of the integrin β1 [24]. Monocytes were treated with MnCl_2_ (5 mM; activation control)_,_ anti-c-myc or anti-CD300a antibodies (5 μg/ml each) for 30 minutes at 37°C. Thereafter, cells were incubated with 15% human serum (to block Fc receptors) in PBS supplemented with 0.1% bovine serum albumin for 10 minutes on ice. Anti-integrin β1 antibodies (9EG7, kindly provided by Prof. Lydia Sorokin) were added for 45 minutes at 4°C, followed by appropriate secondary antibodies and the signals were recorded using a FACS Calibur cytometer (BD Biosciences, Heidelberg, Germany).

### Flow cytometry and internalization

For FACS analysis monocytes (1×10^6^) were incubated with 15% human serum (to block Fc receptors) in PBS supplemented with 0.1% bovine serum albumin for 30 minutes on ice. Thereafter, anti-CD300a or anti-p58.2 antibodies were added followed by appropriate secondary antibodies and the stainings were analysed using a FACS Calibur cytometer (BD Biosciences, Heidelberg, Germany). To analyse total CD300a expresion, cells were permeabilized with cold 70% methanol for 10 minutes at 4°C, followed by blocking and staining as described above. For internalization assays, freshly isolated human monocytes were incubated for different periods of time with anti-CD300a antibodies either at 4°C or at 37°C. Cells were then stained with the appropriate secondary antibodies at 4°C followed by FACS analysis.

### Transfection of COS-7 cells and western blot

COS-7 cells were transfected with the full length CD300a construct together with specific Stealth siRNA oligonucleotides directed at CD300a (5′-UCA CUG CGG CCA AGA CCU CAA CAA U-3′) or control siRNAs (Invitrogen) using Effectene (Qiagen). Two days after transfection, cells were lysed in 0.5% Triton X-100, 10 mM Tris/HCl, pH 7.4, 1 mM MgCl_2_ with protease inhibitors and western blot was performed. The CD300a protein levels were detected using anti-CD300a E59.126 [16].

### Statistics

Statistical comparisons employed Student's t-test; values of *p*<0.05 were regarded as statistically significant.

## Results

### Human peripheral blood monocytes exhibit complex gene expression changes after adhesion and transendothelial migration

To obtain a general overview of monocyte transcriptional regulation associated with the process of adhesion and transendothelial migration, we performed a comprehensive analysis of the gene expression profiles of human peripheral blood monocytes before and after transmigration through an endothelial cell monolayer. The monolayer consisted of human microvascular endothelial cells (HMEC-1) that were seeded on fibronectin-coated polycarbonate filters and used without preactivation by LPS or cytokines/chemokines. Differentially regulated genes were identified by hybridizing cRNA from transmigrated and stationary monocytes to Affymetrix U95Av2 microarrays. On average, 38.2% of the 12599 probe sets found on these microarrays were called present according to the Affymetrix Microarray Suite 5.0 software. Among them, we identified 81 upregulated and 75 downregulated genes showing an average expression change of at least 1.8-fold with p values of less than 0.05 in three independent transmigration and microarray hybridization experiments. The expression changes observed ranged from 14.9-fold down-regulation (IL24) to 9.2-fold up-regulation (VLDLR). A comprehensive list of all genes exhibiting differential expression after adhesion to and migration through an endothelial monolayer is shown in Tables S1 and S2.

In order to confirm the regulations observed in the microarray analysis, we again carried out independent transmigration experiments and subjected 14 of the genes identified in the initial screens to validation by real-time RT-PCR. The genes were chosen to cover a variety of molecular functions, and we only considered the regulation as confirmed if the average regulation in three independent transmigration/RT-PCR experiments matched the direction of regulation observed in the microarray experiments. For 12 of the selected genes we could confirm the direction of regulation found in the microarray experiments (upregulated genes: CD300a, MAP3K8, NT5E, PIK3CB; downregulated genes: AI687419, CCL22, CCR7, FSCN1, GADD45A, RAMP1, RGS2, VNN1) (Table 2). Only the regulation of LILRA2 and PALLD could not be confirmed in the RT-PCR analysis, most likely due to very low absolute expression levels.

**Table 2:** Changes in gene expression of human monocytes induced after transendothelial migration analyzed by realtime RT-PCR.

Accession	Gene symbol	Description	Avg. N-fold (microarray)	Avg. N-fold (RT-PCR)
S67334	PIK3CB	phosphatidylinositol 3-kinase p110 beta isoform = 110 kda catalytic subunit	2.40	1.93
AF020314	CD300A	CD300A antigen	3.56	2.83
X55740	NT5E	5′-nucleotidase, ecto (CD73)	2.05	1.85
U82278	LILRA2	leukocyte immunoglobulin-like receptor, subfamily A (with TM domain), member 2	1.83	−1.05
Z14138	MAP3K8	mitogen-activated protein kinase kinase kinase 8	1.83	1.6
AB023209	PALLD	palladin	−2.48	1.03
AI687419	AI687419	AI687419	−2.93	−3.3
M60974	GADD45A	growth arrest and DNA-damage-inducible, alpha	−2.89	−3.83
U03057	FSCN1	fascin homolog 1, actin-bundling protein (Strongylocentrotus purpuratus)	−2.70	−2.34
U83171	CCL22	chemokine (C-C motif) ligand 22	−2.89	−3.16
L31584	CCR7	chemokine (C-C motif) receptor 7	−2.89	−2.66
L13463	RGS2	regulator of G-protein signalling 2, 24kDa	−3.17	−4.4
AJ132099	VNN1	vanin 1	−2.70	−2.3
AJ001014	RAMP1	receptor (calcitonin) activity modifying protein 1	−7.46	−9.31

### Functional annotation clustering of differentially expressed genes

The genes differentially expressed following monocyte transendothelial migration belong to different functional categories. We therefore subjected the microarray data to further analysis in order to identify biological processes that were statistically overrepresented among these genes using the functional annotation clustering function of the “Database for Annotation, Visualization and Integrated Discovery” (DAVID) 2006 software [25], [26]. Two of the 156 genes found to be up- or downregulated with an expression change of at least 1.8-fold lacked functional gene ontology (GO) annotation and thus could not be considered in the annotation clustering analysis. Using level 4 biological function gene ontology annotation for the remaining 154 genes, DAVID identified two clusters of GO branches containing genes that were statistically overrepresented in comparison to the Affymetrix HG-U95Av2 background gene list. The two clusters contained genes associated with inflammation/immune response (p = 0.001; Table 3) and metabolism (p = 0.016; Table 4), respectively. At least some of the latter are involved in pathways that lead to the production of inflammatory or anti-inflammatory mediators thus showing a relation to inflammatory processes as well.

**Table 3:** Monocyte genes associated with inflammation and immune response found in DAVID functional annotation clustering of the genes differentially expressed following transendothelial migration.

Inflammation/immune response related genes		
Accession	Description	nfold
X68487	adenosine a2b receptor	1.87
D83597	cd180 antigen	4.09
U47924	cd4 antigen (p55)	1.95
M31516	cd55 antigen, decay accelerating factor for complement (cromer blood group)	−1.95
Z11697	cd83 antigen (activated b lymphocytes, immunoglobulin superfamily)	−2.09
U83171	chemokine (c-c motif) ligand 22	−2.89
Y16645	chemokine (c-c motif) ligand 8	−2.19
L31584	chemokine (c-c motif) receptor 7	−2.89
AF054176	chromosome 1 open reading frame 7	1.91
J02931	coagulation factor iii (thromboplastin, tissue factor)	3.73
L12691	defensin, alpha 1	−3.56
X06948	fc fragment of ige, high affinity i, receptor for; alpha polypeptide	−2.05
K03515	glucose phosphate isomerase	2.00
U10550	gtp binding protein overexpressed in skeletal muscle	−9.19
AI660656	immunoglobulin j polypeptide, linker protein for immunoglobulin alpha and mu polypeptides	−2.05
M63438	immunoglobulin kappa constant	−1.95
M34455	indoleamine-pyrrole 2,3 dioxygenase	−2.96
L78833	interferon-induced protein 35	−2.14
AB006537	interleukin 1 receptor accessory protein	3.32
X52015	interleukin 1 receptor antagonist	2.19
U14407	interleukin 15	−2.96
U16261	interleukin 24	−14.93
L19686	macrophage migration inhibitory factor (glycosylation-inhibiting factor)	3.10
M81750	myeloid cell nuclear differentiation antigen	−2.14
S77154	nuclear receptor subfamily 4, group a, member 2	−3.40
U04636	prostaglandin-endoperoxide synthase 2 (prostaglandin g/h synthase and cyclooxygenase)	1.91
J04765	secreted phosphoprotein 1 (osteopontin, bone sialoprotein i, early t-lymphocyte activation 1)	8.98
AL031983	ubiquitin d	−3.91

**Table 4:** Monocyte genes associated with metabolism found in DAVID functional annotation clustering of the genes differentially expressed following transendothelial migration.

Metabolism related genes		
Accession	Description	nfold
D14874	adrenomedullin	2.19
J05032	aspartyl-trna synthetase	2.14
AL021546	cytochrome c oxidase subunit via polypeptide 1	1.82
AF002668	degenerative spermatocyte homolog 1, lipid desaturase (drosophila)	1.87
M55914	enolase 1, (alpha)	1.91
U21931	fructose-1,6-bisphosphatase 1	2.09
L07956	glucan (1,4-alpha-), branching enzyme 1 (glycogen branching enzyme, andersen disease)	2.70
Z12173	glucosamine (n-acetyl)-6-sulfatase (sanfilippo disease iiid)	−1.91
K03515	glucose phosphate isomerase	2.00
D16583	histidine decarboxylase	−1.95
D84424	hyaluronan synthase 1	−2.89
M34455	indoleamine-pyrrole 2,3 dioxygenase	−2.96
X76488	lipase a, lysosomal acid, cholesterol esterase (wolman disease)	1.87
L19686	macrophage migration inhibitory factor (glycosylation-inhibiting factor)	3.10
L22524	matrix metallopeptidase 7 (matrilysin, uterine)	−5.79
D25328	phosphofructokinase, platelet	3.32
M83088	phosphoglucomutase 1	2.52
S81916	phosphoglycerate kinase 1	2.89
J04173	phosphoglycerate mutase 1 (brain)	1.91
L42450	pyruvate dehydrogenase kinase, isozyme 1	2.64
L42452	pyruvate dehydrogenase kinase, isozyme 3	3.17
J05037	serine dehydratase	−3.32
M20681	solute carrier family 2 (facilitated glucose transporter), member 3	2.30
D78130	squalene epoxidase	2.00
U47924	triosephosphate isomerase 1	1.95
X90858	uridine phosphorylase 1	1.95
D16532	very low density lipoprotein receptor	9.19

### Antibody-mediated engagement and downregulation of CD300a affect monocyte transendothelial migration

**Figure 1:**
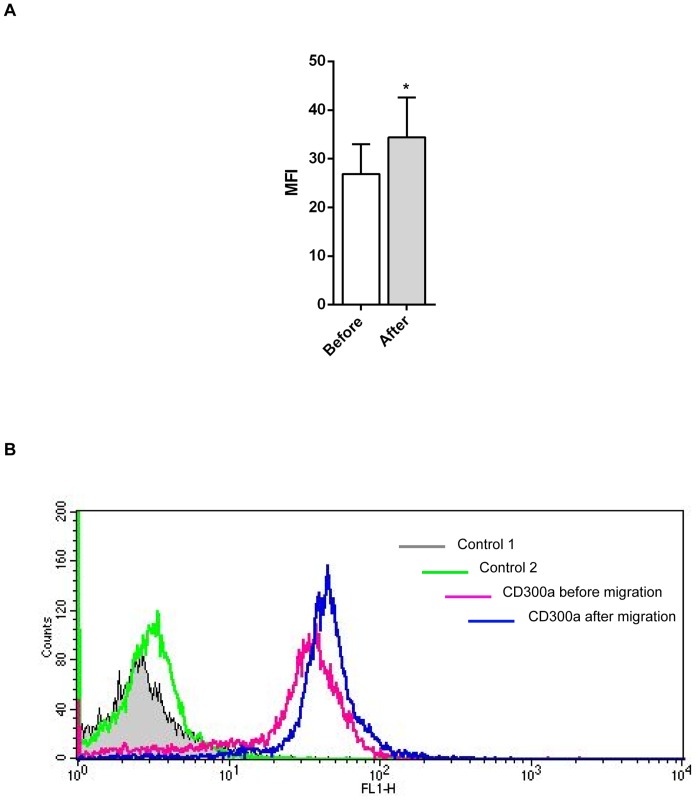
CD300a is upregulated in transmigrated monocytes. Freshly prepared human monocytes were allowed to transmigrate across a monolayer of HMEC1 cells for 4 hours at 37°C, 5% CO_2_. **A**. Mean fluorescence intensity of cell surface CD300a expression obtained by FACS analysis of anti-CD300a antibody stained cells revealing CD300a on the surface of monocytes before and after transendothelial migration. Data presented are from three independent experiments. Statistical significance was evaluated using student's t-test and ratio paired t-test * *P*<0.05. **B**. Representative FACS analysis revealing total CD300a levels before and after transendothelial migration. Control cells were incubated without primary and secondary (control 1) or only with secondary antibodies (control 2).

**Figure 2:**
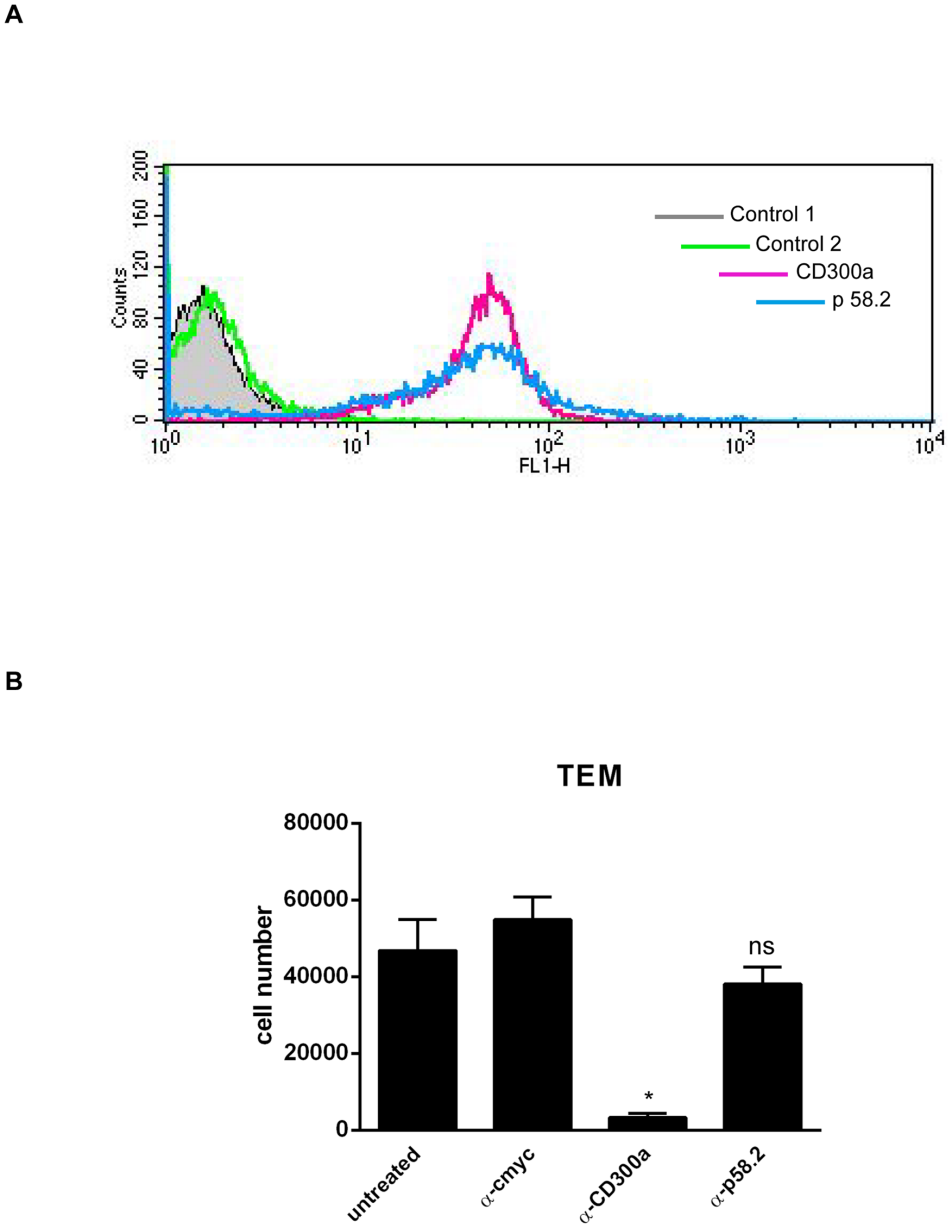
CD300a activation by antibody-engagement on the cell surface of monocytes results in a significant reduction of transendothelial migration. **A**. Cell surface expression of CD300a and p58.2 antigens on human monocytes was analysed by FACS using anti-CD300a and anti-p58.2 antibodies. Control cells were incubated without primary and secondary (control 1) or only with secondary antibodies (control 2). **B**. Freshly isolated human monocytes (2×10^6^) were incubated with mouse monoclonal anti-CD300a (E59.126) or control antibodies (anti-cmyc and anti-p58.2), respectively, and then subjected to transendothelial migration assays in a two-chamber set-up. Migrated cells were collected from the lower chamber and counted using a cell culture analyzer. The number of transmigrated cells is given on the y-axis. Three independent experiments were performed and results are presented as average +/− SEM. Statistical significance was evaluated using student's t-test. * *P*<0.05.

**Figure 3:**
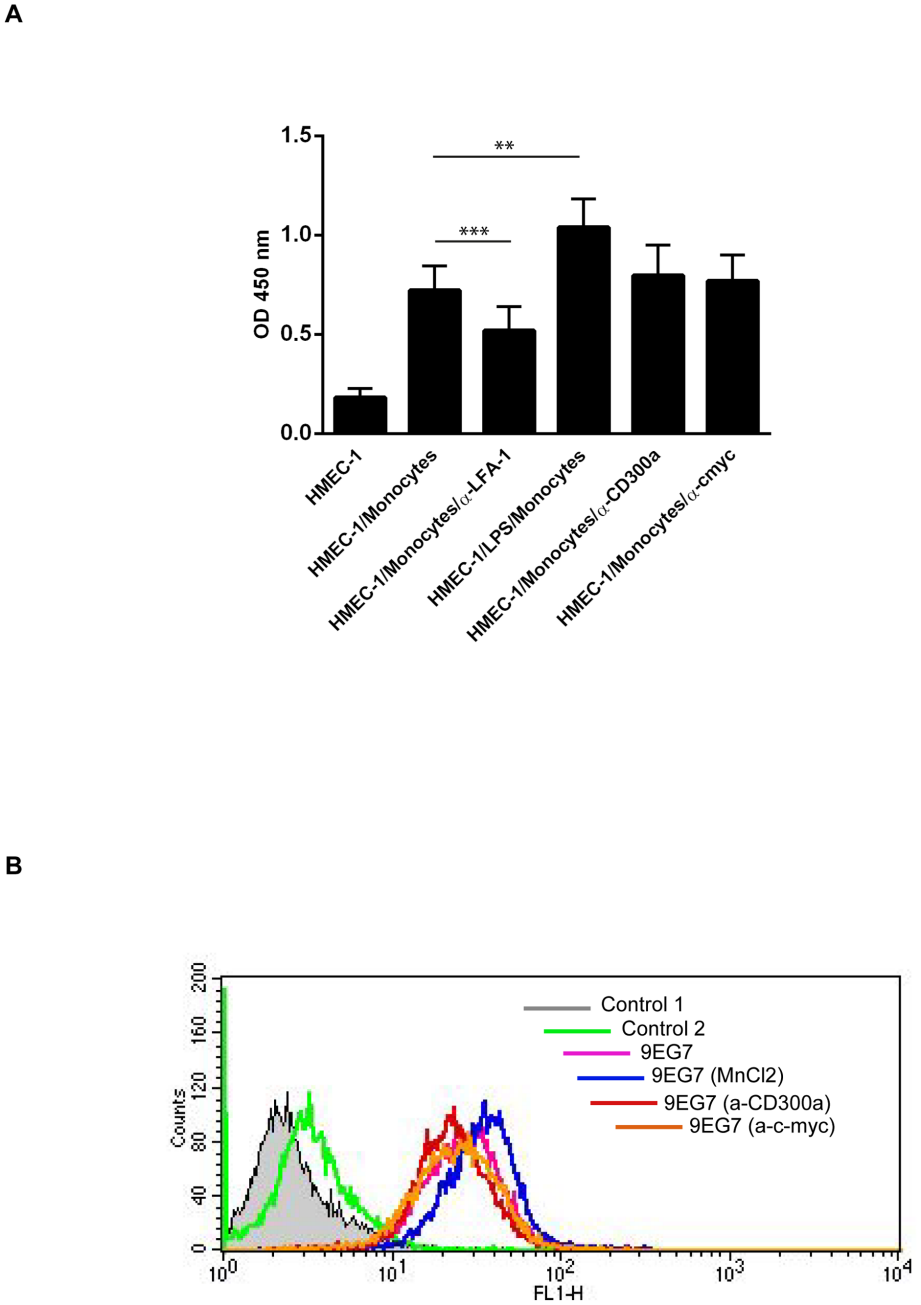
Antibody-engagement of CD300a has no effect on monocyte-endothelial adhesion and integrin activation. **A**. Monocytes were treated with LPS, anti-LFA-1, anti-c-myc or anti-CD300a antibodies and then added to HMEC-1 cells grown in 96 well plates followed by incubation at 37°C. Non-adherent cells were removed by washing with PBS containing Ca++/Mg++ and the amount of adherent cells was determined by measuring the myeloperoxidase activity that is given as OD450 values on the y-axis. Three independent experiments were performed and results are presented as average +/− SEM. Statistical significance was evaluated using student's t-test. ** *P*<0.01 and *** *P*<0.005. **B**. Monocytes were treated first with MnCl_2_ (positive control)_,_ anti-c-myc (negative control) or anti-CD300a antibodies for 30 minutes at 37°C and then at 4°C for 45 minutes with 9EG7 antibodies recognizing the active conformation of β1 integrins. 9EG7 antibodies were then stained with appropriate secondary antibodies followed by FACS analysis.

Our microarray data revealed a significant (about 3.5 fold) upregulation of the mRNA for the inhibitory immune receptor CD300a in migrated as compared to stationary monocytes. To determine whether the increased CD300a mRNA levels also result in elevated cell surface protein levels, we performed FACS analyses of monocytes before and after transendothelial migration employing monoclonal anti-CD300a antibodies. Figure 1A reveals that cell surface CD300a levels are also increased following the transendothelial migration of monocytes. Most likely, this increase is not due to translocation to the cell surface of an existing intracellular pool of the receptor, as the total level of CD300a shows a similar increase after transendothelial migration, indicative of new CD300a protein synthesis (Figure 1B). CD300a is a cell surface receptor of poorly characterized ligand profile and engagement of CD300a by specific antibodies has been shown to trigger receptor phosphorylation and activation in different haematopoetic cell types [16]–[18]. Therefore we employed this protocol to investigate a possible role of CD300a in monocyte transendothelial migration. Freshly isolated human monocytes were incubated with mouse anti-CD300a antibodies and allowed to transmigrate across the HMEC-1 monolayer. Control experiment employed isotype matched mouse IgGs and anti-p58.2 antibodies [18] that were chosen to include another cell surface binding antibody (Figure 2A). Figure 2B reveals that monocytes treated with the anti-CD300a antibody show significantly reduced transmigration as compared to untreated, control IgG-treated or anti-p58.2 treated cells. This effect on transmigration was not due to an altered adhesion capacity of the antibody-treated cells as revealed by quantification of HMEC-1 adherent monocytes in the presence of anti-CD300a versus control antibodies (Fig. 3A). Moreover, anti-CD300a antibody treatment did not affect β-1 integrin activation in monocytes that was visualized by probing with an antibody recognizing an active integrin conformation (9EG7) (Fig. 3B).

**Figure 4:**
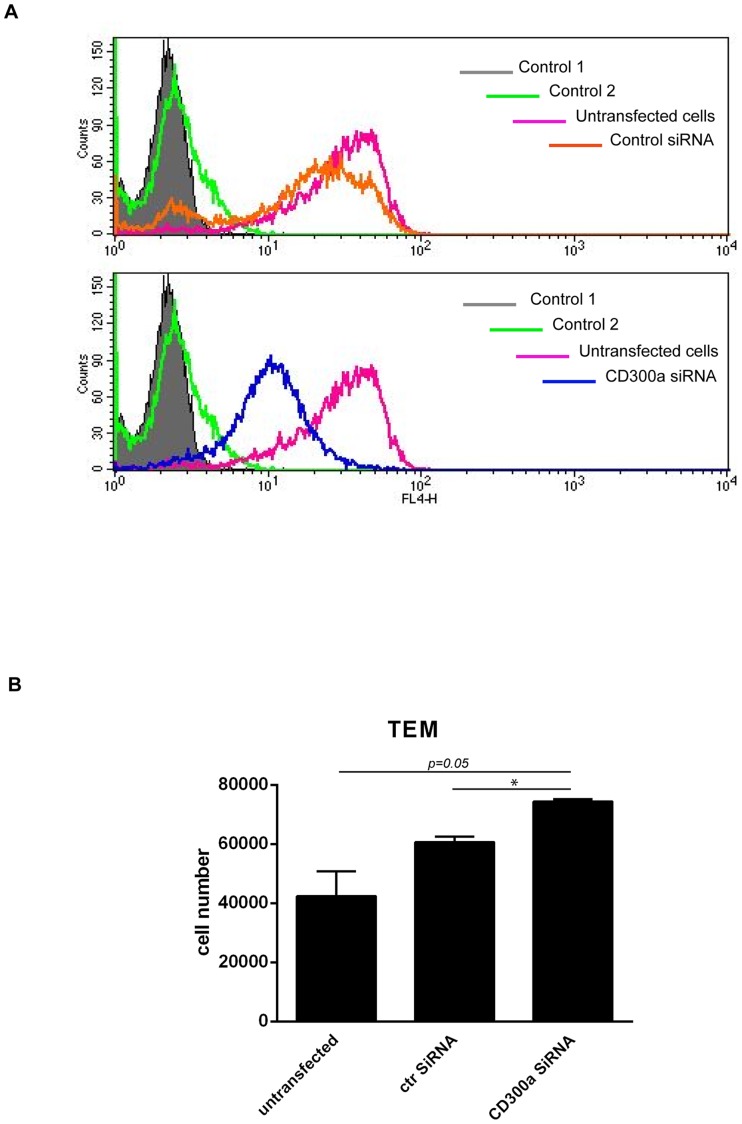
siRNA-mediated downregulation of CD300a increases monocyte transendothelial migration. **A**. FACS analysis of cell surface CD300a expression in monocytes transfected with control or CD300a siRNAs, respectively, employing mouse monoclonal anti-CD300a and appropriately labelled secondary antibodies. Control cells were incubated without primary and secondary (control 1) or only with secondary antibodies (control 2). **B**. Freshly isolated monocytes from human buffy coats were transfected with control or CD300a siRNAs, respectively, followed by transendothelial migration analysis. The number of transmigrated cells is given on the y-axis. Three independent experiments were performed and results are presented as average +/− SEM. Statistical significance was evaluated using student's t-test. * *P*<0.05.

To confirm a functional role of CD300a in regulating the transendothelial migration of monocytes, we used specific siRNA oligonucleotides to knock down CD300a levels. We first verified the efficiency of the siRNA treatment in COS-7 cells that were co-transfected with CD300a-GFP and non-targeting or CD300a-targeting siRNAs, respectively. Western blot analysis of cell lysates prepared 24 h post transfection revealed a significant downregulation of CD300a-GFP in cells transfected with the CD300a-specific siRNA (Figure S1). Next, the efficacy of siRNA-mediated CD300a downregulation was analyzed in monocytes transfected with either control or CD300a-specific siRNAs. Cell surface CD300a was detected by antibody staining two days after transfection revealing a considerable reduction in CD300a levels (Fig. 4A). Given this significant knockdown of surface CD300a, we subjected the CD300a-depleted monocytes to transendothelial migration 48 hours post transfection and compared the number of transmigrated cells to that of untransfected and control siRNA-transfected monocytes. While transfection with control siRNA led to a slight but statistically non-significant increase in transmigration, siRNA-mediated reduction of CD300a cell surface levels resulted in further increase in the number of transmigrated cells that was statistically significant (Fig. 4B).

### Association of CD300a with actin-rich surface structures

**Figure 5:**
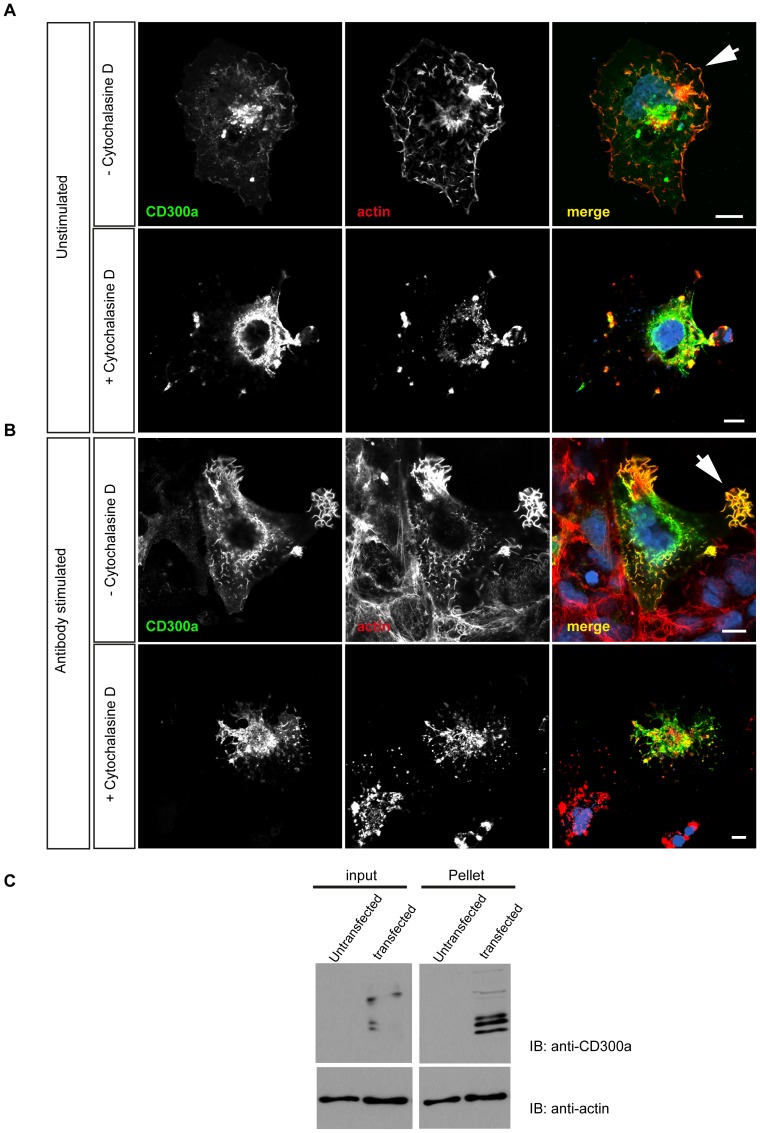
CD300a associates with F-actin in COS-7 cells. **A**. COS-7 cells were transfected with a CD300a expression construct and the subcellular localization of CD300a was then revealed by staining with anti-CD300a antibodies. TRITC-phalloidin was used to label the actin cytoskeleton. The bottom row of images shows cells treated with cytochalasin D (4 μM) to disrupt actin filaments. **B.** COS-7 cells ectopically expressing CD300a were incubated with monoclonal anti-CD300a antibodies to activate cell surface receptors. Cells were then left untreated or treated with 4 μM cytochalasin D followed by fixation and staining with anti-CD300a antibodies and TRITC-phalloidin. Note the localization of CD300a to actin-rich cell protrusions that is increased following the anti-CD300a antibody treatment (arrowhead). Scale bars, 10 μm **C.** Coprecipitation of CD300a with actin filaments. Triton lysates of control (non-transfected) or CD300a expressing COS-7 cells (transfected) were treated with nocodazole to depolymerize microtubules and then subjected to high speed centrifugation to pellet actin filaments. CD300a protein was detected in the actin pellet fraction suggesting a direct or an indirect association of the protein with F-actin. Input shows protein levels in the cell lysates prior to high speed centrifugation.

Monocyte transmigration requires coordinated alterations of cell shape that are mediated by actin cytoskeleton dynamics [27]. Therefore, towards understanding the mechanistic basis of CD300a action in monocyte transmigration we analyzed by subcellular localization studies whether CD300a could directly or indirectly associate with the actin cytoskeleton. Since details of cytoskeletal structures are difficult to visualize in peripheral blood monocytes, experiments were first carried out with COS-7 cells ectopically expressing CD300a. Staining with phalloidin and anti-CD300a antibodies revealed a partial colocalization of CD300a with F-actin, in particular along the cell surface and in actin-rich protrusions (Fig. 5A). It is noteworthy that CD300a localized to such structures but did not induce them, since they are also apparent in untransfected cells. Interestingly, localization of CD300a to actin-rich protrusions was increased following engagment of the receptor with anti-CD300a antibodies. On the other hand, treatment of the cells with cytochalasin D abolished the CD300a- and actin-rich protrusions (Fig. 5B). An interaction of CD300a with F-actin was also evident in actin co-sedimentation assays performed with lysates of cells ectopically expressing CD300a. Figure 5C reveals a significant co-pelleting of CD300a with actin filaments in extracts from transfected cells.

**Figure 6:**
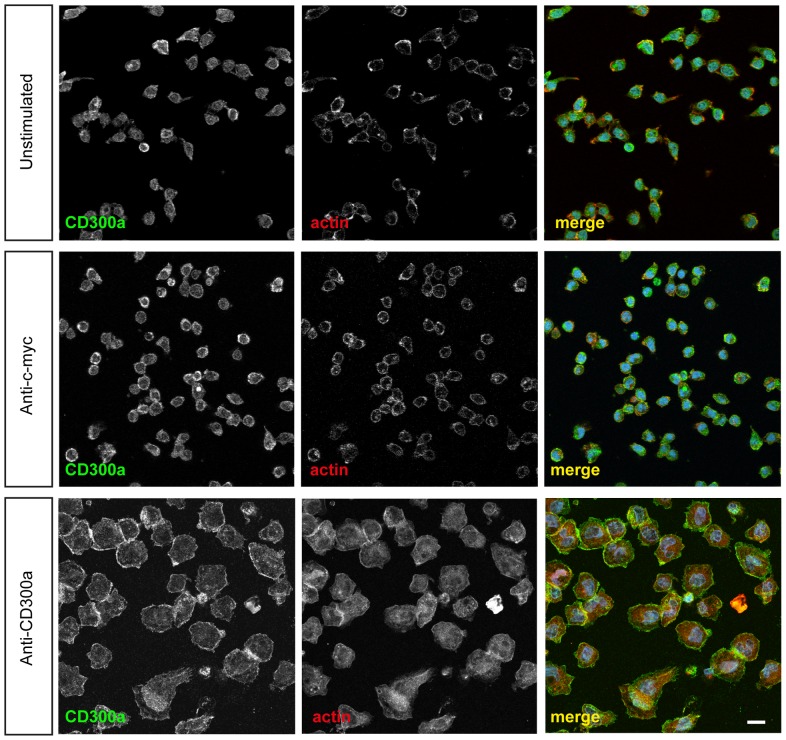
Anti-CD300a antibody treatment alters the morphology of human monocytes. Freshly isolated human monocytes were treated with anti-CD300a IgGs or isotype matched control IgGs (Anti-c-myc) and then seeded on cover slips and incubated at 37°C for an additional 30 min. Cells were then fixed and stained with TRITC-phalloidin and anti-CD300a followed by appropriate secondary antibodies. Scale bars 10 μm.

Given this potential association with the actin cytoskeleton observed in COS-7 cells, we next analyzed whether antibody engagement of CD300a on the surface of human monocytes resulted in F-actin alterations and general morphological changes. Isolated human monocytes were treated with anti-CD300a or isotype-matched control IgGs and then seeded on coverslips for microscopic inspection. Monocytes which were treated with the anti-CD300a antibodies showed a larger and flatter morphology with extended lamellipodia. Isotype-matched control IgGs failed to elicit these morphological changes (Fig. 6).

## Discussion

Transendothelial migration is known to lead to changes in leukocyte biology which enable the cells to function efficiently when reaching the underlying tissue [11]. In line with this, our functional annotation clustering analysis of genes differentially expressed in transmigrated monocytes revealed the highest enrichment score for genes associated with inflammation/immune response. Some of the differentially regulated genes have well-known functions in monocyte-mediated immune responses, such as the interleukin 1 receptor antagonist (IL1RN) (see, for example, [28]. Others, however, are known to play a role in inflammation in general, but have not yet been characterized in monocytes. One such example is CD300a, which is an inhibitory receptor in human NK cells [16] and eosinophils [18] but is also expressed in other leukocytes and was chosen here for further analysis with respect to its potential role in monocyte transmigration.

Our microarray analysis also identified several transcription factors and proteins that modulate transcription factor activity among the differentially expressed genes. These include members of the NR4A nuclear hormone receptor subfamily, NR4A2 and NR4A3, which are known to modulate inflammatory responses [29], and the human myeloid nuclear differentiation antigen (MNDA) [30]. The latter had been implicated in myeloid cell differentiation, although its functional role in this process is unclear [31]. In line with such role of MNDA we obtained additional evidence that transendothelial migration triggered early steps of monocyte differentiation. Comparison of the transcription profile of transmigrated monocytes with that observed upon monocyte-to-macrophage differentiation in phorbol 12-myristate 13-acetate (PMA)-treated U937 cells or cytokine-treated human peripheral blood monocytes [32], [33] revealed that the regulation of several genes, e.g. CCR7, DEFA1, IL1RN, IL15, INDO and SPP1, is common in both scenarios. Thus, altogether our profiling strengthens the hypothesis that human peripheral blood monocytes start differentiating into macrophages already during or immediately after transendothelial migration.

Inhibitory receptors such as CD300a play critical roles in the regulation of different immune cell functions. Loss of inhibitory signalling is often associated with auto-reactivity and excessive inflammatory responses indicating the important role of such inhibitory systems and identifying them as potential targets for the treatment of immune disorders [20], [34]. We show here that CD300a is upregulated during monocyte transendothelial migration not only at the RNA but also at the cell surface protein level. How does this translate to a potential function in monocyte extravasation? In line with an inhibitory role of CD300a, its engagement by specific antibodies inhibits the transendothelial migration of monocytes. This inhibitory effect is similar to that elicited by anti-CD300a antibody engagement on the migration of eosinophils in response to eotaxin [18]. Antibody engagement could induce receptor endocytosis and thereby reduce cell surface levels of CD300a that normally might be required for efficient transmigration. Indeed, the mouse homologue of CD300a (MAIRI/CML-8) contains a YVNL sequence in its cytoplasmic part that is involved in internalization [35] and an internalization motif is also found in the cytoplasmic domain of human CD300a [36]. Therefore, we analysed a possible CD300a internalization following receptor engagement. However, FACS analysis showed no difference in surface expression of CD300a before and after antibody engagement (Figure S2). Thus, we exclude a possible reduction of cell surface CD300a levels in response to antibody treatment in our experiments.

In order to pass through the vessel wall, monocytes need to change their shape, a process accompanied by major cytoskeletal rearrangements [27]. Correlations between inhibitory receptors and actin cytoskeleton dynamics have been reported but are not yet known for CD300a [37]–[39]. Our study reveals for the first time such a relation between CD300a and the actin cytoskeleton. CD300a localizes to actin-rich protrusions that are also positive for ezrin (Figure S3), a protein known to connect membrane receptors to the actin cytoskeleton (for review see [40]). Hence, ezrin might serve as a linker between cell surface CD300a and underlying cortical actin, thereby physically translating CD300a engagement to changes in the actin cytoskeleton. Other, more indirect links between CD300a and the regulation of actin dynamics can also be envisaged. Engagement of CD300a results in phosphorylation of its ITIMs and the recruitment of SHP phosphatases [18]. This could affect actin dynamic because it has been shown that actin is a direct SHP-1 substrate in B cells [41]. BCR crosslinking using specific mAbs results in SHP-1 recruitment and subsequent actin dephosphorylation and F-actin depolymerization. Moreover, Vav1, which is an important regulator of actin dynamics, is also a direct substrate of SHP-1 [42], and its dephosphorylation could represent an important event downstream of CD300a engagement. Alternatively, a physical connection between CD300a and cortical actin might be necessary for the proper recruitment of phosphatases to activated CD300a, similar to what has been observed for FCγRIIB in mast cells [43]. Here, the interaction of FCγRIIB with actin has been shown to be required for enhancing the negative regulatory effect of this receptor towards FCεRI [43]. The role of actin in this case has been assumed to be that of a scaffold protein stabilizing the SHIP-1-FCγRIIB interaction.

As all these receptor-actin interactions appear to negatively regulate cell activities, it is not surprising that antibody engagement of CD300a also has an inhibitory effect on transendothelial migration and that CD300a knockdown increase transmigration. It should be noted, however, that CD300a is upregulated only following adhesion and transendothelial migration and it is likely to act after this step in a physiological scenario. The factor(s) triggering the inhibitory activity of CD300a in this context, i.e. after endothelial passage, remain to be identified. Phosphatidylserine and phosphatidylethanolamine exposed on apoptotic cells have been described recently as CD300a ligands and the receptor appears to be involved in modulating the phagocytosis of dead cells [44], [45]. Thus, it is tempting to speculate that upregulation of monocytic CD300a following transendothelial migration prepares the cells for anti-migratory signalling that is initiated when they get in contact with apoptotic cells at the site of inflammation. Future experiments have to address this hypothesis.

## Supporting Information

Figure S1 Efficient Knockdown of CD300a expression. To analyse the efficiency of CD300a downregulation, COS-7 cells were co-transfected with CD300a-GFP and control or CD300a siRNA duplexes, respectively. Expression of CD300a-GFP was then analysed by immunoblotting of cell lysates using anti-CD300a antibodies. The amount of actin in the cell lysates is shown as a loading control.
(TIF)

Figure S2 Antibody engagement of cell surface CD300a does not result in receptor internalization. Freshly isolated human monocytes were incubated for different periods of time with anti-CD300a antibodies either at 4°C or at 37°C. Thereafter, the amount of cell surface receptor was quantified by FACS analysis as described in Methods. Data are given for 30 and 60 minutes incubation times only. Control cells were incubated without primary and secondary (control 1) or only with secondary antibodies (control 2).
(TIF)

Figure S3 Localization of CD300a in ezrin positive protrusions. Cos-7 cells were transfected with the pcDNA-CD300a construct and stained with rabbit anti-ezrin and mouse anti-CD300a antibodies or phalloidin-TRITC. CD300a localizes to ezrin positive protrusions (arrowhead) which also contain actin.
(TIF)

Table S1 List of monocyte genes upregulated following transendothelial migration.
(DOC)

Table S2 List of monocyte genes downregulated following transendothelial migration.
(DOC)

## References

[1] Choi EY, Santoso S, Chavakis T (2009) Mechanisms of neutrophil transendothelial migration. Front Biosci 14: 1596–1605. 10.2741/3327 PMC2672407 19273149

[2] Ley K, Laudanna C, Cybulsky MI, Nourshargh S (2007) Getting to the site of inflammation: the leukocyte adhesion cascade updated. Nat Rev Immunol 7: 678–689. 17717539 10.1038/nri2156

[3] Muller WA (2011) Mechanisms of Leukocyte Transendothelial Migration. Annual Review of Pathology: Mechanisms of Disease 6: 323–344. 10.1146/annurev-pathol-011110-130224 PMC3628537 21073340

[4] van Buul JD, Hordijk PL (2004) Signaling in Leukocyte Transendothelial Migration. Arteriosclerosis, Thrombosis, and Vascular Biology 24: 824–833. 10.1161/01.ATV.0000122854.76267.5c 14976004

[5] Woodfin A, Voisin M-B, Nourshargh S (2010) Recent developments and complexities in neutrophil transmigration. Current Opinion in Hematology 17: 9–17. 19864945 10.1097/MOH.0b013e3283333930 PMC2882030

[6] Imhof BA, Aurrand-Lions M (2004) Adhesion mechanisms regulating the migration of monocytes. Nat Rev Immunol 4: 432–444. 15173832 10.1038/nri1375

[7] Zarbock A, Ley K, McEver RP, Hidalgo As (2011) Leukocyte ligands for endothelial selectins: specialized glycoconjugates that mediate rolling and signaling under flow. Blood 118: 6743–6751. 22021370 10.1182/blood-2011-07-343566 PMC3245201

[8] de Fougerolles AR, Chi-Rosso G, Bajardi A, Gotwals P, Green CD, et al (2000) Global Expression Analysis of Extracellular Matrix-Integrin Interactions in Monocytes. Immunity 13: 749–758. 11163191 10.1016/s1074-7613(00)00073-x

[9] Rossetti G, Collinge M, Molteni R, Bender JR, Pardi R (2002) Integrin-dependent regulation of gene expression in leukocytes. Immunological Reviews 186: 189–207. 12234372 10.1034/j.1600-065x.2002.18616.x

[10] Shi C, Simon DI (2006) Integrin Signals, Transcription Factors, and Monocyte Differentiation. Trends in Cardiovascular Medicine 16: 146–152. 16781947 10.1016/j.tcm.2006.03.002

[11] Nourshargh S, Marelli-Berg FM (2005) Transmigration through venular walls: a key regulator of leukocyte phenotype and function. Trends in Immunology 26: 157–165. 15745858 10.1016/j.it.2005.01.006

[12] Srivastava M, Jung S, Wilhelm J, Fink L, Bühling F, et al (2005) The Inflammatory versus Constitutive Trafficking of Mononuclear Phagocytes into the Alveolar Space of Mice Is Associated with Drastic Changes in Their Gene Expression Profiles. The Journal of Immunology 175: 1884–1893. 16034132 10.4049/jimmunol.175.3.1884

[13] Thomas-Ecker S, Lindecke A, Hatzmann W, Kaltschmidt C, Zänker KS, et al (2007) Alteration in the gene expression pattern of primary monocytes after adhesion to endothelial cells. Proceedings of the National Academy of Sciences 104: 5539–5544. 10.1073/pnas.0700732104 PMC1838499 17372200

[14] Moretta A, Bottino C, Vitale M, Pende D, Cantoni C, et al (2001) Activating receptors and coreceptors involved in human natural killer cell-mediated cytolysis. Annual Review of Immunology 19: 197–223. 10.1146/annurev.immunol.19.1.197 11244035

[15] Moretta L, Moretta A (2004) Unravelling natural killer cell function: triggering and inhibitory human NK receptors. EMBO J 23: 255–259. 14685277 10.1038/sj.emboj.7600019 PMC1271745

[16] Cantoni C, Bottino C, Augugliaro R, Morelli L, Marcenaro E, et al (1999) Molecular and functional characterization of IRp60, a member of the immunoglobulin superfamily that functions as an inhibitory receptor in human NK cells. European Journal of Immunology 29: 3148–3159. 10540326 10.1002/(SICI)1521-4141(199910)29:10<3148::AID-IMMU3148>3.0.CO;2-L

[17] Bachelet I, Munitz A, Moretta A, Moretta L, Levi-Schaffer F (2005) The Inhibitory Receptor IRp60 (CD300a) Is Expressed and Functional on Human Mast Cells. The Journal of Immunology 175: 7989–7995. 16339535 10.4049/jimmunol.175.12.7989

[18] Munitz A, Bachelet I, Eliashar R, Moretta A, Moretta L, et al (2006) The inhibitory receptor IRp60 (CD300a) suppresses the effects of IL-5, GM-CSF, and eotaxin on human peripheral blood eosinophils. Blood 107: 1996–2003. 16254138 10.1182/blood-2005-07-2926

[19] Alvarez Y, Tang X, Coligan JE, Borrego F (2008) The CD300a (IRp60) inhibitory receptor is rapidly up-regulated on human neutrophils in response to inflammatory stimuli and modulates CD32a (FcγRIIa) mediated signaling. Molecular Immunology 45: 253–258. 17588661 10.1016/j.molimm.2007.05.006 PMC2000843

[20] Bachelet I, Munitz A, Berent-Maoz B, Mankuta D, Levi-Schaffer F (2008) Suppression of Normal and Malignant Kit Signaling by a Bispecific Antibody Linking Kit with CD300a. The Journal of Immunology 180: 6064–6069. 18424727 10.4049/jimmunol.180.9.6064

[21] Denholm EM, Wolber FM (1991) A simple method for the purification of human peripheral blood monocytes. A substitute for Sepracell-MN. J Immunol Methods 144: 247–251. 1960422 10.1016/0022-1759(91)90092-t

[22] Kielbassa K, Schmitz C, Gerke V (1998) Disruption of Endothelial Microfilaments Selectively Reduces the Transendothelial Migration of Monocytes. Experimental Cell Research 243: 129–141. 9716457 10.1006/excr.1998.4133

[23] Livak KJ, Schmittgen TD (2001) Analysis of Relative Gene Expression Data Using Real-Time Quantitative PCR and the 2-Delta Delta CT Method. Methods 25: 402–408. 11846609 10.1006/meth.2001.1262

[24] Lenter M, Uhlig H, Hamann A, Jenö P, Imhof B, et al (1993) A monoclonal antibody against an activation epitope on mouse integrin chain beta 1 blocks adhesion of lymphocytes to the endothelial integrin alpha 6 beta 1. Proceedings of the National Academy of Sciences 90: 9051–9055. 10.1073/pnas.90.19.9051 PMC47499 7692444

[25] Dennis G Jr, Sherman BT, Hosack DA, Yang J, Gao W, et al (2003) DAVID: Database for Annotation, Visualization, and Integrated Discovery. Genome Biol 4: P3. 12734009 PMC193660

[26] Hosack DA DGJ, Sherman BT, Lane HC, Lempicki RA (2003) Identifying biological themes within lists of genes with EASE. Genome Biol 4: R70. 14519205 10.1186/gb-2003-4-10-r70 PMC328459

[27] Weber KSC, Klickstein LB, Weber PC, Weber C (1998) Chemokine-induced monocyte transmigration requires cdc42-mediated cytoskeletal changes. European Journal of Immunology 28: 2245–2251. 9692894 10.1002/(SICI)1521-4141(199807)28:07<2245::AID-IMMU2245>3.0.CO;2-V

[28] Arend WP, Malyak M, Guthridge CJ, Gabay C (1998) Interlukin-1 receptor antagonist: Role in Biology. Annual Review of Immunology 16: 27–55. 10.1146/annurev.immunol.16.1.27 9597123

[29] Bonta PI, van Tiel CM, Vos M, Pols TWH, van Thienen JV, et al (2006) Nuclear Receptors Nur77, Nurr1, and NOR-1 Expressed in Atherosclerotic Lesion Macrophages Reduce Lipid Loading and Inflammatory Responses. Arteriosclerosis, Thrombosis, and Vascular Biology 26: 2288. 10.1161/01.ATV.0000238346.84458.5d 16873729

[30] Briggs RC, Atkinson JB, Miranda RN (2005) Variable expression of human myeloid specific nuclear antigen MNDA in monocyte lineage cells in atherosclerosis. Journal of Cellular Biochemistry 95: 293–301. 15778972 10.1002/jcb.20435

[31] Asefa B, Klarmann KD, Copeland NG, Gilbert DJ, Jenkins NA, et al (2004) The interferon-inducible p200 family of proteins: a perspective on their roles in cell cycle regulation and differentiation. Blood Cells, Molecules, and Diseases 32: 155–167. 10.1016/j.bcmd.2003.10.002 14757431

[32] Martinez FO, Gordon S, Locati M, Mantovani A (2006) Transcriptional Profiling of the Human Monocyte-to-Macrophage Differentiation and Polarization: New Molecules and Patterns of Gene Expression. The Journal of Immunology 177: 7303–7311. 17082649 10.4049/jimmunol.177.10.7303

[33] Verhoeckx KCM, Bijlsma S, de Groene EM, Witkamp RF, van der Greef J, et al (2004) A combination of proteomics, principal component analysis and transcriptomics is a powerful tool for the identification of biomarkers for macrophage maturation in the U937 cell line. Proteomics 4: 1014–1028. 15048983 10.1002/pmic.200300669

[34] Tam SW, Demissie S, Thomas D, Daëron M (2004) A bispecific antibody against human IgE and human FcγRII that inhibits antigen-induced histamine release by human mast cells and basophils. Allergy 59: 772–780. 15180766 10.1111/j.1398-9995.2004.00332.x

[35] Yotsumoto K, Okoshi Y, Shibuya K, Yamazaki S, Tahara-Hanaoka S, et al (2003) Paired activating and inhibitory immunoglobulin-like receptors, MAIR-I and MAIR-II, regulate mast cell and macrophage activation. J Exp Med 198: 223–233. 12874256 10.1084/jem.20021825 PMC2194075

[36] Clark GJ, Ju X, Tate C, Hart DNJ (2009) The CD300 family of molecules are evolutionarily significant regulators of leukocyte functions. Trends in Immunology 30: 209–217. 19359216 10.1016/j.it.2009.02.003

[37] Krzewski K, Chen X, Orange JS, Strominger JL (2006) Formation of a WIP-, WASp-, actin-, and myosin IIA-containing multiprotein complex in activated NK cells and its alteration by KIR inhibitory signaling. The Journal of Cell Biology 173: 121–132. 16606694 10.1083/jcb.200509076 PMC2063796

[38] Masilamani M, Nguyen C, Kabat J, Borrego F, Coligan JE (2006) CD94/NKG2A Inhibits NK Cell Activation by Disrupting the Actin Network at the Immunological Synapse. The Journal of Immunology 177: 3590–3596. 16951318 10.4049/jimmunol.177.6.3590

[39] Toyama-Sorimachi N, Tsujimura Y, Maruya M, Onoda A, Kubota T, et al (2004) Ly49Q, a member of the Ly49 family that is selectively expressed on myeloid lineage cells and involved in regulation of cytoskeletal architecture. Proceedings of the National Academy of Sciences of the United States of America 101: 1016–1021. 14732700 10.1073/pnas.0305400101 PMC327143

[40] Fehon RG, McClatchey AI, Bretscher A (2010) Organizing the cell cortex: the role of ERM proteins. Nat Rev Mol Cell Biol 11: 276–287. 20308985 10.1038/nrm2866 PMC2871950

[41] Baba T, Fusaki N, Shinya N, Iwamatsu A, Hozumi N (2003) Actin Tyrosine Dephosphorylation by the Src Homology 1-Containing Protein Tyrosine Phosphatase Is Essential for Actin Depolymerization After Membrane IgM Cross-Linking. The Journal of Immunology 170: 3762–3768. 12646642 10.4049/jimmunol.170.7.3762

[42] Stebbins CC, Watzl C, Billadeau DD, Leibson PJ, Burshtyn DN, et al (2003) Vav1 Dephosphorylation by the Tyrosine Phosphatase SHP-1 as a Mechanism for Inhibition of Cellular Cytotoxicity. Molecular and Cellular Biology 23: 6291–6299. 12917349 10.1128/MCB.23.17.6291-6299.2003 PMC180957

[43] Lesourne R, Fridman WH, Daeron M (2005) Dynamic Interactions of Fc gamma Receptor IIB with Filamin-Bound SHIP1 Amplify Filamentous Actin-Dependent Negative Regulation of Fc epsilon Receptor I Signaling. The Journal of Immunology 174: 1365–1373. 15661894 10.4049/jimmunol.174.3.1365

[44] Nakahashi-Oda C, Tahara-Hanaoka S, Shoji M, Okoshi Y, Nakano-Yokomizo T, et al (2012) Apoptotic cells suppress mast cell inflammatory responses via the CD300a immunoreceptor. The Journal of Experimental Medicine 209: 1493–1503. 22826299 10.1084/jem.20120096 PMC3409498

[45] Simhadri VR, Andersen JF, Calvo E, Choi S-C, Coligan JE, et al.. (2012) Human CD300a binds to phosphatidylethanolamine and phosphatidylserine, and modulates the phagocytosis of dead cells. Blood 119 2799–2809. 10.1182/blood-2011-08-372425 PMC3327458 22302738

