# Supplementary figures and images for: Transcriptional Profiling of Human Monocytes Identifies the Inhibitory Receptor CD300a as Regulator of Transendothelial Migration
Source: PLoS One. 2013 Sep 18;8(9):e73981. doi: 10.1371/journal.pone.0073981 (PMC3776808; doi:10.1371/journal.pone.0073981)

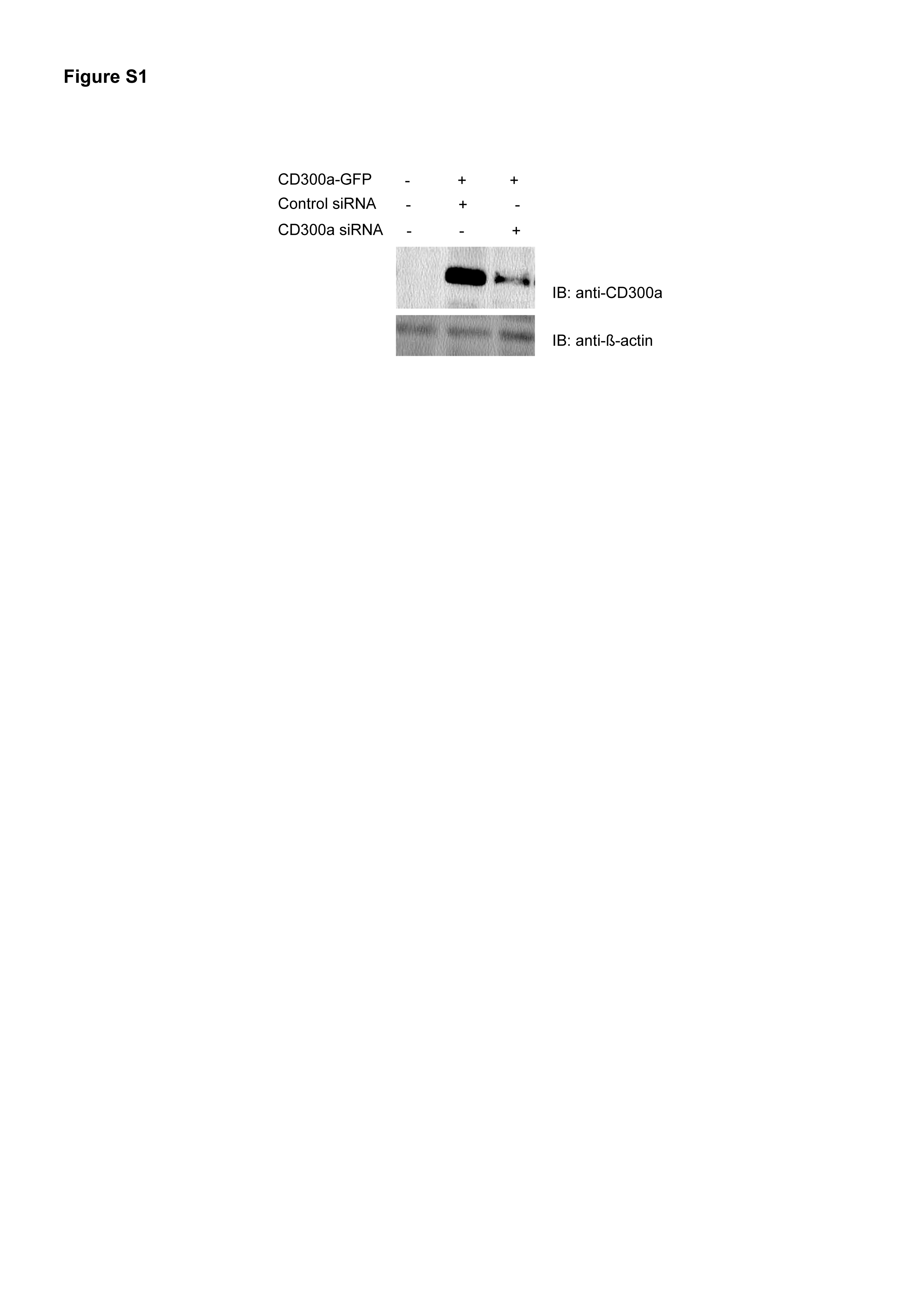

Supplement: Figure S1 — Efficient Knockdown of CD300a expression. To analyse the efficiency of CD300a downregulation, COS-7 cells were co-transfected with CD300a-GFP and control or CD300a siRNA duplexes, respectively. Expression of CD300a-GFP was then analysed by immunoblotting of cell lysates using anti-CD300a antibodies. The amount of actin in the cell lysates is shown as a loading control. (TIF) [file pone.0073981.s001.tif]

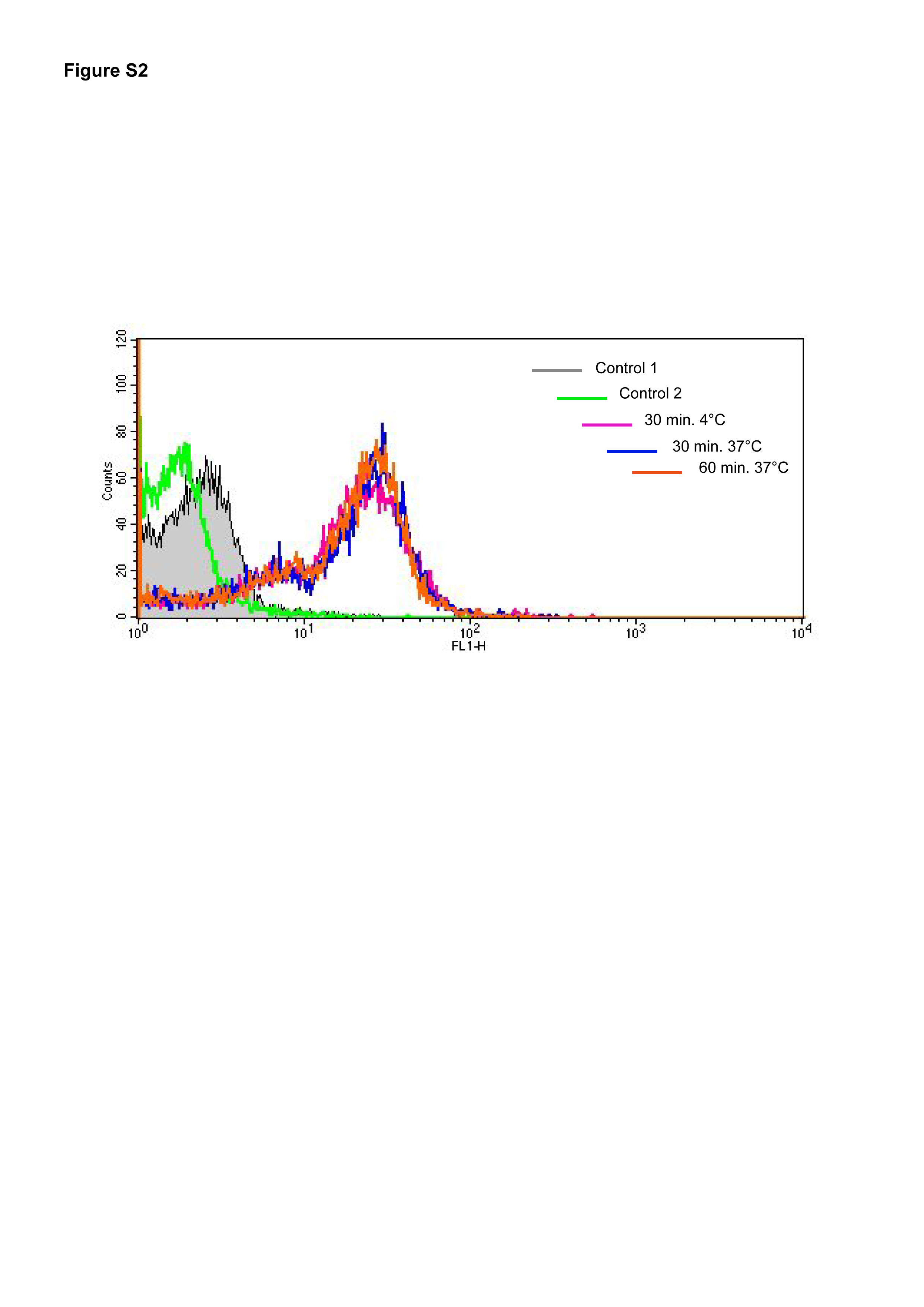

Supplement: Figure S2 — Antibody engagement of cell surface CD300a does not result in receptor internalization. Freshly isolated human monocytes were incubated for different periods of time with anti-CD300a antibodies either at 4°C or at 37°C. Thereafter, the amount of cell surface receptor was quantified by FACS analysis as described in Methods. Data are given for 30 and 60 minutes incubation times only. Control cells were incubated without primary and secondary (control 1) or only with secondary antibodies (control 2). (TIF) [file pone.0073981.s002.tif]

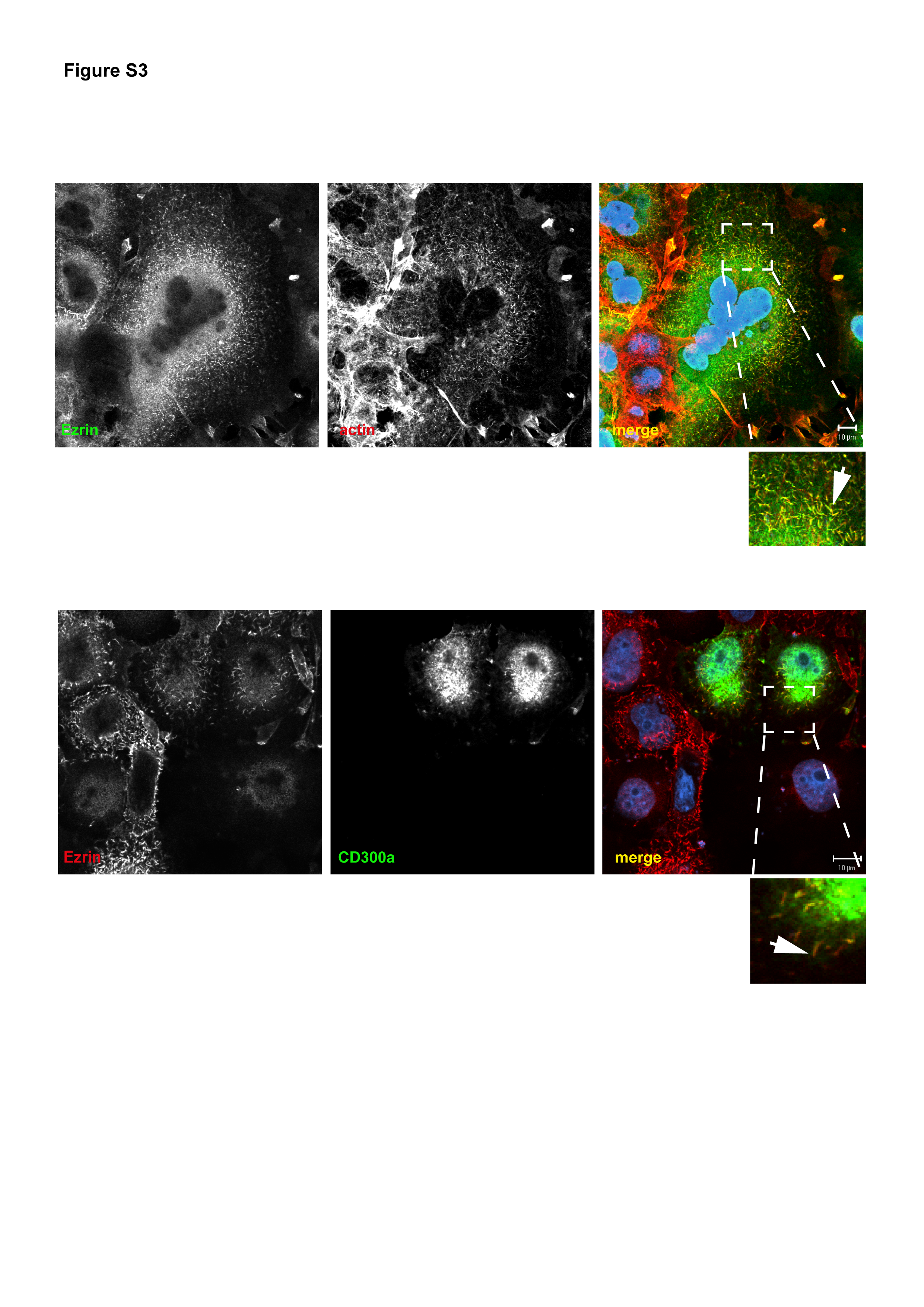

Supplement: Figure S3 — Localization of CD300a in ezrin positive protrusions. Cos-7 cells were transfected with the pcDNA-CD300a construct and stained with rabbit anti-ezrin and mouse anti-CD300a antibodies or phalloidin-TRITC. CD300a localizes to ezrin positive protrusions (arrowhead) which also contain actin. (TIF) [file pone.0073981.s003.tif]
