# Supplementary material for: Transcriptional Profiling of Human Monocytes Identifies the Inhibitory Receptor CD300a as Regulator of Transendothelial Migration
Source: PLoS One. 2013 Sep 18;8(9):e73981. doi: 10.1371/journal.pone.0073981 (PMC3776808; doi:10.1371/journal.pone.0073981)
Supplement: Table S1 — List of monocyte genes upregulated following transendothelial migration. (DOC) [file pone.0073981.s004.doc]

**Supplemental Table S1. List of monocyte genes upregulated following transendothelial migration**

| **Accesion** | **Gene symbol** | **Description** | **nFold** |
| --- | --- | --- | --- |
| D16532 | VLDLR | very low density lipoprotein receptor | 9,19 |
| J04765 | SPP1 | secreted phosphoprotein 1 (osteopontin, bone sialoprotein I, early T-lymphocyte activation 1) | 8,98 |
| D83597 | CD180 | CD180 antigen | 4,09 |
| J02931 | F3 | coagulation factor III (thromboplastin, tissue factor) | 3,73 |
| AF020314 | CD300A | CD300A antigen | 3,56 |
| X97324 | ADFP | adipose differentiation-related protein | 3,48 |
| AB006537 | IL1RAP | interleukin 1 receptor accessory protein | 3,32 |
| D25328 | PFKP | phosphofructokinase, platelet | 3,32 |
| AI038821 | HRAS | v-Ha-ras Harvey rat sarcoma viral oncogene homolog | 3,17 |
| L42452 | PDK3 | pyruvate dehydrogenase kinase, isoenzyme 3 | 3,17 |
| AF002697 | BNIP3 | BCL2/adenovirus E1B 19kDa interacting protein 3 | 3,17 |
| L19686 | MIF | macrophage migration inhibitory factor (glycosylation-inhibiting factor) | 3,10 |
| X51956 | ENO2 | enolase 2 (gamma, neuronal) | 3,10 |
| S81916 | PGK1 | phosphoglycerate kinase 1 | 2,89 |
| AA926957 | FLJ10534 | hypothetical protein FLJ10534 | 2,83 |
| AL080235 | RIS1 | Ras-induced senescence 1 | 2,70 |
| L07956 | GBE1 | glucan (1,4-alpha-), branching enzyme 1 | 2,70 |
| L42450 | PDK1 | pyruvate dehydrogenase kinase, isoenzyme 1 | 2,64 |
| D87953 | NDRG1 | N-myc downstream regulated gene 1 | 2,64 |
| M24486 | P4HA1 | procollagen-proline, 2-oxoglutarate 4-dioxygenase (proline 4-hydroxylase), alpha polypeptide I | 2,64 |
| AF055993 | SAP30 | sin3-associated polypeptide, 30kDa | 2,52 |
| M83088 | PGM1 | phosphoglucomutase 1 | 2,52 |
| AF050110 | KLF10 | Kruppel-like factor 10 | 2,52 |
| AB023207 | CHSY1 | carbohydrate (chondroitin) synthase 1 | 2,52 |
| S73591 | TXNIP | thioredoxin interacting protein | 2,46 |
| X80821 | ANKRD12 | ankyrin repeat domain 12 | 2,46 |
| U84573 | PLOD2 | procollagen-lysine, 2-oxoglutarate 5-dioxygenase 2 | 2,41 |
| S67334 | PIK3CB | phosphatidylinositol 3-kinase p110 beta isoform=110 kda catalytic subunit | 2,40 |
| AL021977 | MAFF | v-maf musculoaponeurotic fibrosarcoma oncogene homolog F (avian) | 2,35 |
| M20681 | SLC2A3 | solute carrier family 2 (facilitated glucose transporter), member 3 | 2,30 |
| U25182 | PRDX4 | peroxiredoxin 4 | 2,30 |
| AJ131244 | SEC24A | SEC24 related gene family, member A (S. cerevisiae) | 2,24 |
| X52015 | IL1RN | interleukin 1 receptor antagonist | 2,19 |
| D14874 | ADM | adrenomedullin | 2,19 |
| AB018285 | JMJD1A | jumonji domain containing 1A | 2,14 |
| J05032 | DARS | aspartyl-tRNA synthetase | 2,14 |
| U21931 | FBP1 | fructose-1,6-bisphosphatase 1 | 2,09 |
| D88827 | ZNF263 | zinc finger protein 263 | 2,05 |
| AC004982 | FLJ20323 | hypothetical protein FLJ20323 | 2,05 |
| AF052288 | HIP1 | huntingtin interacting protein 1 | 2,05 |
| X55740 | NT5E | 5'-nucleotidase, ecto (CD73) | 2,05 |
| AF024710 | VEGF | vascular endothelial growth factor | 2,00 |
| D78130 | SQLE | squalene epoxidase | 2,00 |
| AB002345 | PER2 | period homolog 2 (Drosophila) | 2,00 |
| K03515 | GPI | glucose phosphate isomerase | 2,00 |
| M69199 | G0S2 | G0/G1switch 2 | 2,00 |
| X90858 | UPP1 | uridine phosphorylase 1 | 1,95 |
| M37721 | PAM | peptidylglycine alpha-amidating monooxygenase | 1,95 |
| X51758 | HSPA6 | heat shock 70kDa protein 6 (HSP70B') | 1,95 |
| U47924 | TPI1 | triosephosphate isomerase 1 | 1,95 |
| D14041 | RBPSUH | recombining binding protein suppressor of hairless (Drosophila) | 1,95 |
| U50928 | PKD2 | polycystic kidney disease 2 (autosomal dominant) | 1,95 |
| AB007870 | NUPL1 | nucleoporin like 1 | 1,95 |
| U04636 | PTGS2 | prostaglandin-endoperoxide synthase 2 (prostaglandin G/H synthase and cyclooxygenase) | 1,91 |
| J04173 | PGAM1 | phosphoglycerate mutase 1 (brain) | 1,91 |
| AF079221 | BNIP3L | BCL2/adenovirus E1B 19kDa interacting protein 3-like | 1,91 |
| AJ002428 | VDAC1 | voltage-dependent anion channel 1 | 1,91 |
| AI557497 | UBE2J1 | ubiquitin-conjugating enzyme E2, J1 (UBC6 homolog, yeast) | 1,91 |
| AL049246 | SLC25A36 | Solute carrier family 25, member 36 | 1,91 |
| U48250 | OLIG2 | oligodendrocyte lineage transcription factor 2 | 1,91 |
| M55914 | ENO1 | enolase 1, (alpha) | 1,91 |
| AF054176 | CIAS1 | cold autoinflammatory syndrome 1 | 1,91 |
| W26496 | WSB1 | WD repeat and SOCS box-containing 1 | 1,87 |
| AB015718 | STK10 | serine/threonine kinase 10 | 1,87 |
| L48692 | LOC56902 | putatative 28 kDa protein | 1,87 |
| X76488 | LIPA | lipase A, lysosomal acid, cholesterol esterase (Wolman disease) | 1,87 |
| AF002668 | DEGS1 | degenerative spermatocyte homolog 1, lipid desaturase (Drosophila) | 1,87 |
| U41387 | DDX21 | DEAD (Asp-Glu-Ala-Asp) box polypeptide 21 | 1,87 |
| M57763 | ARF6 | ADP-ribosylation factor 6 | 1,87 |
| X68487 | ADORA2B | adenosine A2b receptor | 1,87 |
| U82278 | LILRA2 | leukocyte immunoglobulin-like receptor, subfamily A (with TM domain), member 2 | 1,83 |
| Z14138 | MAP3K8 | mitogen-activated protein kinase kinase kinase 8 | 1,83 |
| M27878 | ZNF84 | zinc finger protein 84 (HPF2) | 1,82 |
| X77744 | ZNF160 | zinc finger protein 160 | 1,82 |
| X95762 | XPNPEP1 | X-prolyl aminopeptidase (aminopeptidase P) 1, soluble | 1,82 |
| J04111 | JUN | v-jun sarcoma virus 17 oncogene homolog (avian) | 1,82 |
| AL021546 | SFRS9 | splicing factor, arginine/serine-rich 9 | 1,82 |
| AB007858 | RNMT | RNA (guanine-7-) methyltransferase | 1,82 |
| AL080184 | INSIG2 | insulin induced gene 2 | 1,82 |
| U07550 | HSPE1 | heat shock 10kDa protein 1 (chaperonin 10) | 1,82 |
| AL096713 | FER1L3 | fer-1-like 3, myoferlin (C. elegans) | 1,82 |
| AB004066 | BHLHB2 | basic helix-loop-helix domain containing, class B, 2 | 1,82 |
| X60674 | AK3L1 | adenylate kinase 3-like 1 | 1,82 |
| AF017789 | TCERG1 | transcription elongation regulator 1 | 1,82 |
