# Supplementary material for: Transcriptional Profiling of Human Monocytes Identifies the Inhibitory Receptor CD300a as Regulator of Transendothelial Migration
Source: PLoS One. 2013 Sep 18;8(9):e73981. doi: 10.1371/journal.pone.0073981 (PMC3776808; doi:10.1371/journal.pone.0073981)
Supplement: Table S2 — List of monocyte genes downregulated following transendothelial migration. (DOC) [file pone.0073981.s005.doc]

**Supplemental Table S2. List of monocyte genes downregulated following transendothelial migration**

| **Accesion** | **Gene symbol** | **Description** | **nFold** |
| --- | --- | --- | --- |
| U16261 | IL24 | interleukin 24 | -14,93 |
| U10550 | GEM | GTP binding protein overexpressed in skeletal muscle | -9,19 |
| M27826 | psiTPTE22 | TPTE pseudogene | -7,82 |
| AJ001014 | RAMP1 | receptor (calcitonin) activity modifying protein 1 | -7,46 |
| U42408 | LAD1 | ladinin 1 | -5,79 |
| L22524 | MMP7 | matrix metallopeptidase 7 (matrilysin, uterine) | -5,79 |
| U22662 | NR1H3 | nuclear receptor subfamily 1, group H, member 3 | -4,81 |
| M60315 | BMP6 | bone morphogenetic protein 6 | -4,59 |
| AA128249 | FABP4 | fatty acid binding protein 4, adipocyte | -4,49 |
| L01694 | GNA12 | guanine nucleotide binding protein (G protein) alpha 12 | -4,49 |
| AL031983 | UBD | ubiquitin D | -3,91 |
| L12691 | DEFA1 | defensin, alpha 1 | -3,56 |
| U63289 | CUGBP1 | CUG triplet repeat, RNA binding protein 1 | -3,48 |
| S77154 | NR4A2 | nuclear receptor subfamily 4, group A, member 2 | -3,40 |
| U18088 | PDE4A | phosphodiesterase 4A, cAMP-specific (phosphodiesterase E2 dunce homolog, Drosophila) | -3,32 |
| J05037 | SDS | serine dehydratase | -3,32 |
| L13463 | RGS2 | regulator of G-protein signalling 2, 24kDa | -3,17 |
| L13740 | NR4A1 | nuclear receptor subfamily 4, group A, member 1 | -3,10 |
| U14407 | IL15 | interleukin 15 | -2,96 |
| M34455 | INDO | indoleamine-pyrrole 2,3 dioxygenase | -2,96 |
| AI687419 | AI687419 | AI687419 | -2.93 |
| U83171 | CCL22 | chemokine (C-C motif) ligand 22 | -2,89 |
| L31584 | CCR7 | chemokine (C-C motif) receptor 7 | -2,89 |
| M60974 | GADD45A | growth arrest and DNA-damage-inducible, alpha | -2,89 |
| D84424 | HAS1 | hyaluronan synthase 1 | -2,89 |
| AL049332 | BTG3 | BTG family, member 3 | -2,76 |
| U12767 | NR4A3 | nuclear receptor subfamily 4, group A, member 3 | -2,76 |
| U03057 | FSCN1 | fascin homolog 1, actin-bundling protein (Strongylocentrotus purpuratus) | -2,70 |
| AJ132099 | VNN1 | vanin 1 | -2,70 |
| AB013924 | LAMP3 | lysosomal-associated membrane protein 3 | -2,58 |
| M86904 | DMD | dystrophin (muscular dystrophy, Duchenne and Becker types) | -2,52 |
| U60644 | PLD3 | phospholipase D family, member 3 | -2,52 |
| X79780 | RAB11B | RAB11B, member RAS oncogene family | -2,52 |
| AB023209 | PALLD | palladin | -2,48 |
| AF017786 | PPAP2B | phosphatidic acid phosphatase type 2B | -2,46 |
| X52056 | SPI1 | spleen focus forming virus (SFFV) proviral integration oncogene spi1 | -2,41 |
| AC005390 | KIAA0963 | KIAA0963 | -2,35 |
| L19871 | ATF3 | activating transcription factor 3 | -2,35 |
| S71043 | IGHA1 | immunoglobulin heavy constant alpha 1 | -2,35 |
| U12471 | THBS1 | thrombospondin 1 | -2,24 |
| Y16645 | CCL8 | chemokine (C-C motif) ligand 8 | -2,19 |
| AB011175 | TBC1D4 | TBC1 domain family, member 4 | -2,19 |
| AB011143 | GAB2 | GRB2-associated binding protein 2 | -2,19 |
| M81750 | MNDA | myeloid cell nuclear differentiation antigen | -2,14 |
| L78833 | VAT1 | vesicle amine transport protein 1 homolog (T californica) | -2,14 |
| L76380 | CALCRL | calcitonin receptor-like | -2,09 |
| M73720 | CPA3 | carboxypeptidase A3 (mast cell) | -2,09 |
| Z11697 | CD83 | CD83 antigen (activated B lymphocytes, immunoglobulin superfamily) | -2,09 |
| U22398 | CDKN1C | cyclin-dependent kinase inhibitor 1C (p57, Kip2) | -2,09 |
| AB026436 | DUSP10 | dual specificity phosphatase 10 | -2,09 |
| S76638 | NFKB2 | nuclear factor of kappa light polypeptide gene enhancer in B-cells 2 (p49/p100) | -2,09 |
| X06948 | FCER1A | Fc fragment of IgE, high affinity I, receptor for; alpha polypeptide | -2,05 |
| X51435 | HIVEP1 | human immunodeficiency virus type I enhancer binding protein 1 | -2,05 |
| AI660656 | IGJ | Immunoglobulin J polypeptide, linker protein for immunoglobulin alpha and mu polypeptides | -2,05 |
| Y12696 | CLIC2 | chloride intracellular channel 2 | -2,00 |
| L37882 | FZD2 | frizzled homolog 2 (Drosophila) | -2,00 |
| X79783 | IGLC1 | immunoglobulin lambda constant 1 (Mcg marker) | -2,00 |
| L32976 | MAP3K11 | mitogen-activated protein kinase kinase kinase 11 | -2,00 |
| U77735 | PIM2 | pim-2 oncogene | -2,00 |
| M31516 | DAF | decay accelerating factor for complement (CD55, Cromer blood group system) | -1,95 |
| AF078077 | GADD45B | growth arrest and DNA-damage-inducible, beta | -1,95 |
| D16583 | HDC | histidine decarboxylase | -1,95 |
| X98248 | KLHDC3 | kelch domain containing 3 | -1,95 |
| AF037989 | SOCS2 | suppressor of cytokine signaling 2 | -1,95 |
| M63438 | IGKC | immunoglobulin kappa constant /// immunoglobulin kappa variable 1-5 | -1,95 |
| Z12173 | GNS | glucosamine (N-acetyl)-6-sulfatase (Sanfilippo disease IIID) | -1,91 |
| AL049471 | ARID5B | AT rich interactive domain 5B (MRF1-like) | -1,91 |
| X05323 | CD200 | CD200 antigen | -1,91 |
| X05610 | COL4A2 | collagen, type IV, alpha 2 | -1,91 |
| L22473 | BAX | BCL2-associated X protein | -1,87 |
| J02958 | MET | met proto-oncogene (hepatocyte growth factor receptor) | -1,87 |
| U44103 | RAB9A | RAB9A, member RAS oncogene family | -1,87 |
| AA521060 | ABHD5 | Abhydrolase domain containing 5 | -1,82 |
| AF007130 | MDM1 | Mdm4, transformed 3T3 cell double minute 1, p53 binding protein (mouse) | -1,82 |
| Z35102 | STK38 | serine/threonine kinase 38 | -1,82 |
| U68494 | SLC30A1 | Solute carrier family 30 (zinc transporter), member 1 | -1,82 |
| U97669 | NOTCH3 | Notch homolog 3 (Drosophila) | -1,82 |
